# A Comparison of Outcomes in Patients with Secondary Acute Promyelocytic Leukemia (APL) and De Novo APL—A Case-Matched Retrospective Analysis of the Polish Adult Leukemia Group (PALG)

**DOI:** 10.3390/cancers18182899

**Published:** 2026-09-08

**Authors:** Agnieszka Pluta, Damian Mikulski, Marta Sobas, Kinga Strzałka, Magdalena Czemerska, Dominika Trybunia-Orzeszek, Tomasz Wrobel, Bozena Budziszewska, Marzena Wątek, Ewa Lech-Maranda, Tomasz Gromek, Dorota Hawrylecka, Marek Hus, Ewa Zarzycka, Jan Maciej Zaucha, Anna Armatys, Grzegorz Helbig, Jolanta Oleksiuk, Łukasz Bołkun, Andrzej Szczepaniak, Lidia Gil, Rafał Becht, Wojciech Fendler, Sebastian Giebel, Agnieszka Wierzbowska

**Affiliations:** 1 Department of Hematology, Medical University of Lodz, 90-419 Lodz, Polandagawierzbowska@wp.pl (A.W.); 2 The Nicolas Copernicus Multidisciplinary Provincial Center of Traumatology and Oncology, 93-513 Lodz, Poland; 3 Department of Biostatistics and Translational Medicine, Medical University of Lodz, 93-510 Lodz, Poland; 4 Department of Hematology, Blood Neoplasm and Bone Marrow Transplantation, Piast Medical University, 50-367 Wroclaw, Poland; 5 Department of Hematology, Collegium Medicum in Bydgoszcz, Nicolaus Copernicus University in Torun, 85-094 Bydgoszcz, Poland; 6 Department of Hematology, Institute of Hematology and Transfusion Medicine, 02-776 Warsaw, Polandmarzena.watek@wp.pl (M.W.);; 7 Department of Hematooncology and Bone Marrow Transplantation, Medical University of Lublin, 20-080 Lublin, Poland; 8 Department of Hematology and Transplantation, Medical University of Gdansk, 80-952 Gdansk, Poland; 9 Department of Hematology and Bone Marrow Transplantation, Medical School of Silesia, 40-032 Katowice, Polandghelbig@o2.pl (G.H.); 10 Department of Hematology, Medical University Hospital, 15-276 Bialystok, Poland; 11 Department of Hematology, Transplantation and Cellular Therapy, Poznan University of Medical Sciences, 60-569 Poznan, Poland; 12 Clinical Department of Oncology, Chemotherapy and Cancer Immunotherapy, Pomeranian Medical University, 71-252 Szczecin, Poland; 13 Department of Bone Marrow Transplantation and Onco-Hematology, Maria Sklodowska-Curie Institute–Cancer Center, Gliwice Branch, 44-101 Gliwice, Poland

**Keywords:** de novo APL, secondary APL, treatment, CD15

## Abstract

Background: Secondary acute promyelocytic leukemia (sAPL) is a very rare subtype of acute myeloid leukemia that develops following exposure to chemotherapy, radiotherapy, or immunosuppressive agents. The treatment results and survival outcomes of sAPL patients are still not precisely defined. Methods: A search of the Polish Adult Leukemia Group (PALG) database identified 29 cases of sAPL (median age 57 years; 62.1% female) among 437 APL patients (6.7%) diagnosed between 2006 and 2024. Each sAPL case was matched to a de novo APL patient by sex, age, year of diagnosis, and treatment protocol (LPA (Leucemia Promielocítica Aguda) 2005, LPA 2012, or LPA 2017). Results: All sAPL cases occurred following chemo- and/or radiotherapy, most commonly for breast cancer. The sAPL cases demonstrated higher CD15 expression than the de novo APL cases (median 27.6% vs. 7%, *p* = 0.04). The two groups exhibited comparable complete remission rates (82.8% sAPL vs. 75.9% de novo; *p* = 0.75) and early mortality rates (17.2% vs. 20.7%, *p* = 1.0). However, relapse-free survival was significantly shorter in sAPL (median 94.7 months vs. not reached; HR 7.23, 95% CI 1.63–32.02, *p* = 0.030), whereas overall survival did not differ significantly between groups. Multivariate analysis identified Eastern Cooperative Oncology Group performance status ≥3 and CD15 expression > 20% as independent predictors of inferior survival. Conclusions: These findings suggest that sAPL shares many clinical features with de novo APL but carries a higher risk of relapse, highlighting the need for further prospective studies and the potential implementation of tailored therapeutic strategies.

## Simple Summary

Secondary acute promyelocytic leukemia (sAPL) is a very rare subtype of AML that can develop in patients previously treated with chemotherapy or radiotherapy. It has remained unclear whether these patients respond to treatment and survive as well as patients who develop de novo APL. Using the Polish Adult Leukemia Group (PALG) database, we compared twenty-nine patients with secondary disease to a matched group with de novo APL. Both groups achieved complete remission at similar rates. However, patients with sAPL relapsed more often and showed higher levels of the cell-surface marker called CD15, which is linked to poorer survival. These findings suggest that, although sAPL can be treated successfully, patients may benefit from closer monitoring and tailored treatment strategies to decrease their risk of relapse.

## 1. Introduction

Acute promyelocytic leukemia (APL) is a rare subtype of acute myeloid leukemia, characterized by t(15;17) (q24;q21) translocation and the presence of the *PML-RARα* fusion gene [1,2]. The RARECAREnet database indicates that the incidence of APL in Europe is 0.112 per 100,000 population per year (95% CI, 0.107–0.117) [3,4]. While the majority of APL cases arise de novo, a 5–15% manifest following exposure to genotoxic agents such as anthracyclines, topoisomerase II inhibitors, immunosuppression or radiotherapy, such cases are known as secondary APL (sAPL). While de novo APL has a favorable prognosis, only limited data exists regarding sAPL, although a few studies have found sAPL patients to have a shorter median overall survival (OS) than de novo APL patients [4,5,6,7]. Nevertheless, sAPL patients still demonstrate better long-term outcomes than those with other types of secondary acute myeloid leukemia (sAML) [8,9,10,11,12,13], with some studies suggesting that the two have comparable outcomes [8,9,14].

Recent advances in APL-specific therapies, particularly the use of all-trans retinoic acid (ATRA) and arsenic trioxide (ATO), have significantly improved survival in de novo APL, raising the question of whether similar benefits can be achieved in sAPL. This question remains unclear as, due to the rarity of sAPL, it has been the subject of little research to date.

Therefore, the aim of this analysis was to compare the outcomes of patients with sAPL to those of matched cases with de novo APL. It also determines whether sAPL exhibits a different outcome.

## 2. Materials and Methods

The PALG database of APL patients from 2006 to 2024 was screened to identify cases of sAPL. All identified patients with sAPL were manually matched 1:1, without replacement, to patients with de novo APL from the PALG database. Exact agreement was required for sex, calendar year of diagnosis, and treatment protocol, while age was matched using a maximum permitted within-pair difference of three years. These four matching criteria were considered together, without propensity-score matching or another automated optimization algorithm. When more than one control fulfilled the matching criteria, one patient was selected manually from the eligible candidates, and each control was included in only one matched pair. After the matched cohort had been defined, participating sites were contacted to complete missing clinical and outcome data for the selected patients.

All patients had been treated according to three consecutive PETHEMA protocols for APL. In the LPA 2005 protocol, the induction consisted of oral ATRA (45 mg/m^2^/d), given until complete remission (CR), in combination with idarubicin (IDA) at 12 mg/m^2^/d i.v. on days 2, 4, 6, and 8 (AIDA regimen). Consolidation, based on ATRA and anthracycline, was adjusted according to predefined risk groups. The patients in the low-risk group (WBC ≤ 10 × 10^3^/L and platelet count > 40 × 10^3^/L) and the intermediate-risk group (WBC ≤ 10 × 10^3^/L and platelet count ≤ 40 × 10^3^/L) received a reduced dose of mitoxantrone during the second consolidation course, whereas high-risk patients (WBC > 10 × 10^3^/L) younger than 60 years received cytarabine combined with ATRA and idarubicin during the first and third consolidation courses. After consolidation, maintenance treatment consisting of ATRA combined with 6-mercaptopurine and methotrexate was scheduled for two years in accordance with the applicable PETHEMA protocol [15].

The second protocol (LPA 2012) used the same treatment schema as in LPA 2005; however, patients with CD56 expression greater than 20% were classified into a higher-risk group. The results of this treatment have not yet been published. The third protocol, LPA 2017, was created based on the results of the Lo-Coco et al. trial [16]. In this trial, patients with a WBC < 10 × 10^3^/L received an ATRA and ATO regimen for induction and consolidation, whereas those with higher WBC counts received standard AIDA induction and consolidation treatment. For the high-risk group, a maintenance therapy, as described above, was administered [16,17]. The treatment protocols are described in more detail in the pivotal trials [15,16].

### Statistical Analysis

The distribution of continuous variables was determined using the Shapiro–Wilk test. Baseline characteristics were compared using the Mann–Whitney U-test for continuous variables and the Chi-square test for categorical variables; Yates’ correction or Fisher’s exact tests were applied where appropriate. Univariate and multivariate Cox regression models were constructed to evaluate the impact of clinical and laboratory variables on survival. Median follow-up was estimated using the reverse Kaplan–Meier method, in which deaths were treated as censored observations and patients alive at the last assessment were treated as events. Follow-up distributions between the sAPL and de novo APL groups were compared using the log-rank test. Kaplan–Meier curves were generated to visualize survival differences, and comparisons between curves were performed using the log-rank test. All analyses were conducted using Statistica 13.3 software (TIBCO, Palo Alto, CA, USA) and GraphPad Prism 8.0.2.

## 3. Results

Out of 437 patients in the database, 29 patients with sAPL (6.70%) were identified. Most were female (*n* = 18, 62.07%), and their median age was 57 years (IQR: 44–60). The clinical characteristics of the study groups are given in Table 1.

### 3.1. Primary Disease Overview

All reported cases of sAPL occurred after previous exposure to chemo- and/or radiotherapy. Among the sAPL patients, disease onset followed neoplasm treatment in 28 cases (96.55%), and multiple sclerosis therapy in the remaining case (3.45%). sAPL was most often diagnosed following treatment for breast cancer (*n* = 8, 26.6%), although some cases were noted after endometrial (*n* = 4, 13.32%), testicular (*n* = 2, 6.66%), lung (*n* = 2, 6.66%) and colon cancers (*n* = 2, 6.66%). In individual cases, sAPL followed other forms of cancer: kidney cancer, liposarcoma, laryngeal cancer, ovarian cancer, Hodgkin lymphoma, B-acute lymphoblastic leukemia, Merkel cell tumor or gastrointestinal stromal tumor (GIST). In another two cases, the primary site of the neoplasm could not be determined. The median time from initial diagnosis of malignancy to sAPL was 56 months (95% CI: 38.00–135.43).

### 3.2. Laboratory Findings and Clinical Presentation at the Time of APL Diagnosis

Generally speaking, no significant differences in clinical and laboratory characteristics were observed between sAPL and de novo APL (Table 1). However, the sAPL group tended to have a lower platelet count at diagnosis (median 26.5 G/L, IQR: 16–54 vs. 37 G/L, IQR: 25–57) and was more likely to present ECOG stage ≥ 3 (22.2% vs. 6.9%); however, neither difference was statistically significant (*p* = 0.19 and *p* = 0.14, respectively). The sAPL patients exhibited significantly greater CD15 expression compared to de novo APL (median 27.6%, IQR: 2.43–51.50 vs. 7%, IQR: 0.62–26.00, *p* = 0.04) (Supplementary Figure S1). They were also more frequently CD15 positive (>20%) than de novo APL (48.3% sAPL vs. 34.5% de novo); however, the difference was not statistically significant. No significant differences in the frequency of additional cytogenetic abnormalities (20.7% vs. 27.6%; *p* = 0.21) or *FLT3-ITD* co-mutation (28.6% and 29.4%; *p* = 1.0) were observed between the two groups.

Bleeding at diagnosis was more common in sAPL (66.7% vs. 44.8%, *p* = 0.19) than in de novo APL. Both groups were characterized mostly by mucocutaneous bleedings; in addition, one case of orbital hematoma was observed in the sAPL group, and one intraocular hemorrhage and one bleeding from the lower gastrointestinal tract in the de novo group. Similarly, no significant difference in the frequency of thrombotic complications was found between the groups (sAPL: 11.5% vs. de novo APL: 20.7%, *p* = 0.47).

### 3.3. Treatment Outcome

All APL patients were treated according to PETHEMA protocols for APL: LPA 2005 (*n* = 36), LPA 2012 (*n* = 12), LPA 2017 (*n* = 10) [11,12,16].

The incidence of differentiation syndrome (DS) did not differ significantly between the sAPL and de novo APL groups (19.1% vs. 32%; *p* = 0.5), neither did CR rate after induction (sAPL 82.8% vs. de novo APL 75.9%, *p* = 0.75) nor early mortality, i.e., up to 30 days (sAPL 17.2% vs. 20.7%, *p* = 1.0).

Using the reverse Kaplan–Meier method, the median follow-up was 31.5 months for the overall cohort, 30.7 months for the sAPL group, and 31.5 months for the de novo APL group. Follow-up duration did not differ significantly between the groups (log-rank *p* = 0.722).

**Figure 1:**
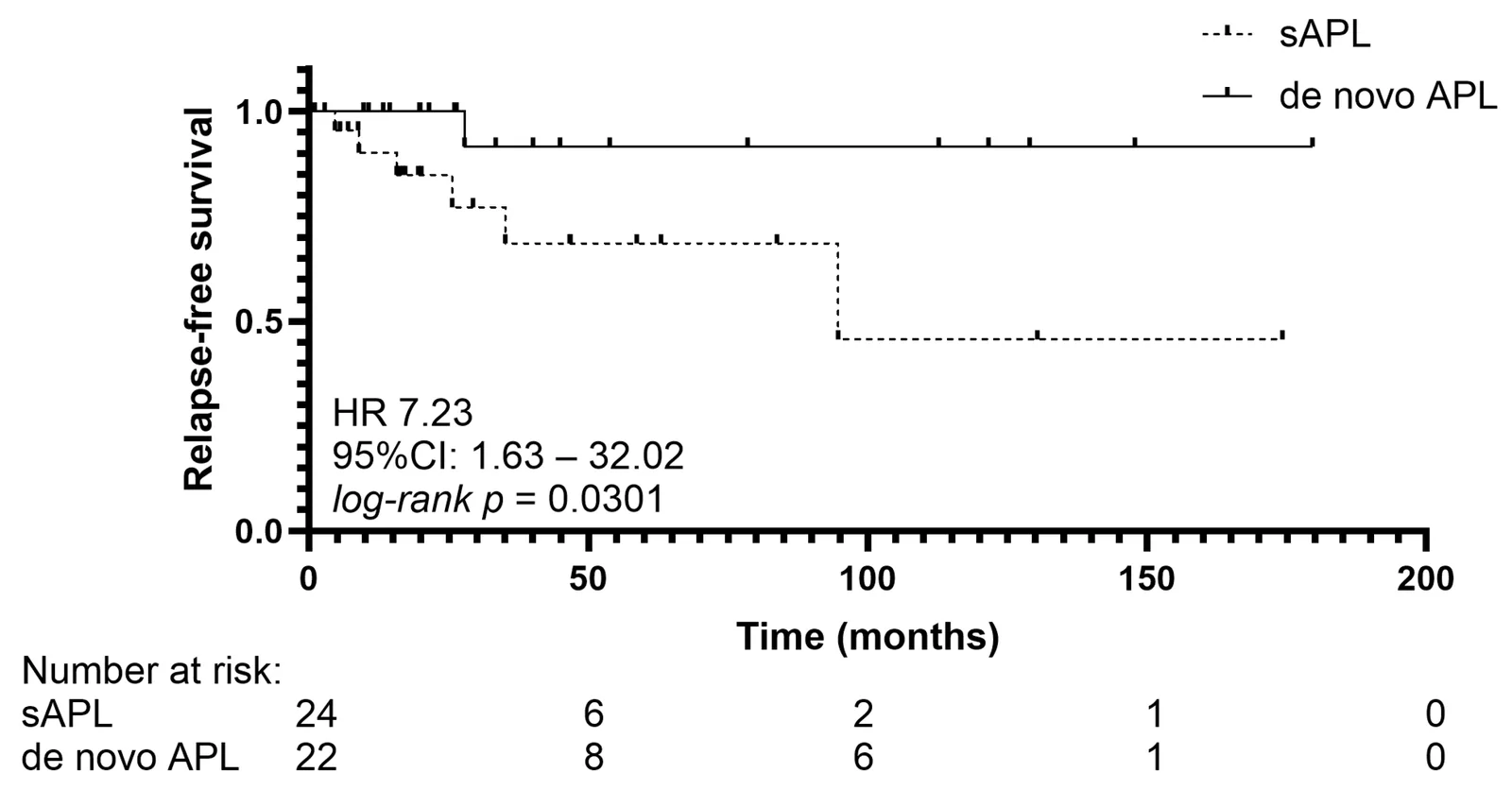
A comparison of relapse-free survival (RFS) between secondary APL and de novo APL. APL—acute promyelocytic leukemia; sAPL—secondary APL.

Overall, four (16.7%) patients in the sAPL group and one (4.35%) in the de novo APL group relapsed. The median relapse-free survival (RFS) was not reached (NR) for the APL group as a whole. However, the sAPL patients had significantly shorter RFS (HR 7.23, 95% CI: 1.63–32.02) compared to de novo APL: median RFS was 94.7 months in the sAPL group (95% CI: 35.1 -NR) but was not reached in the de novo APL group (*p* = 0.030) (Figure 1).

The single patient with relapsed de novo APL received ATO–ATRA and intrathecal treatment for combined hematological and extramedullary relapse and was alive in CR2 at the last recorded follow-up. Among the four patients with sAPL who relapsed, one received IDA–ATRA reinduction, achieved CR2, and subsequently received ATO–ATRA followed by autologous HSCT (autoHSCT). One achieved CR2 after ATO–ATRA regimen, followed by autoHSCT, and subsequently died in a second APL relapse. One patient with CNS relapse received intrathecal methotrexate, cytarabine, and dexamethasone and died shortly thereafter. Salvage treatment was not documented for one patient who died from hemorrhage 21 days after relapse. Overall, CR2 was explicitly documented in three patients. At the database cutoff, the relapsed patient with de novo APL was alive, whereas all four patients with relapsed sAPL were recorded as deceased.

**Figure 2:**
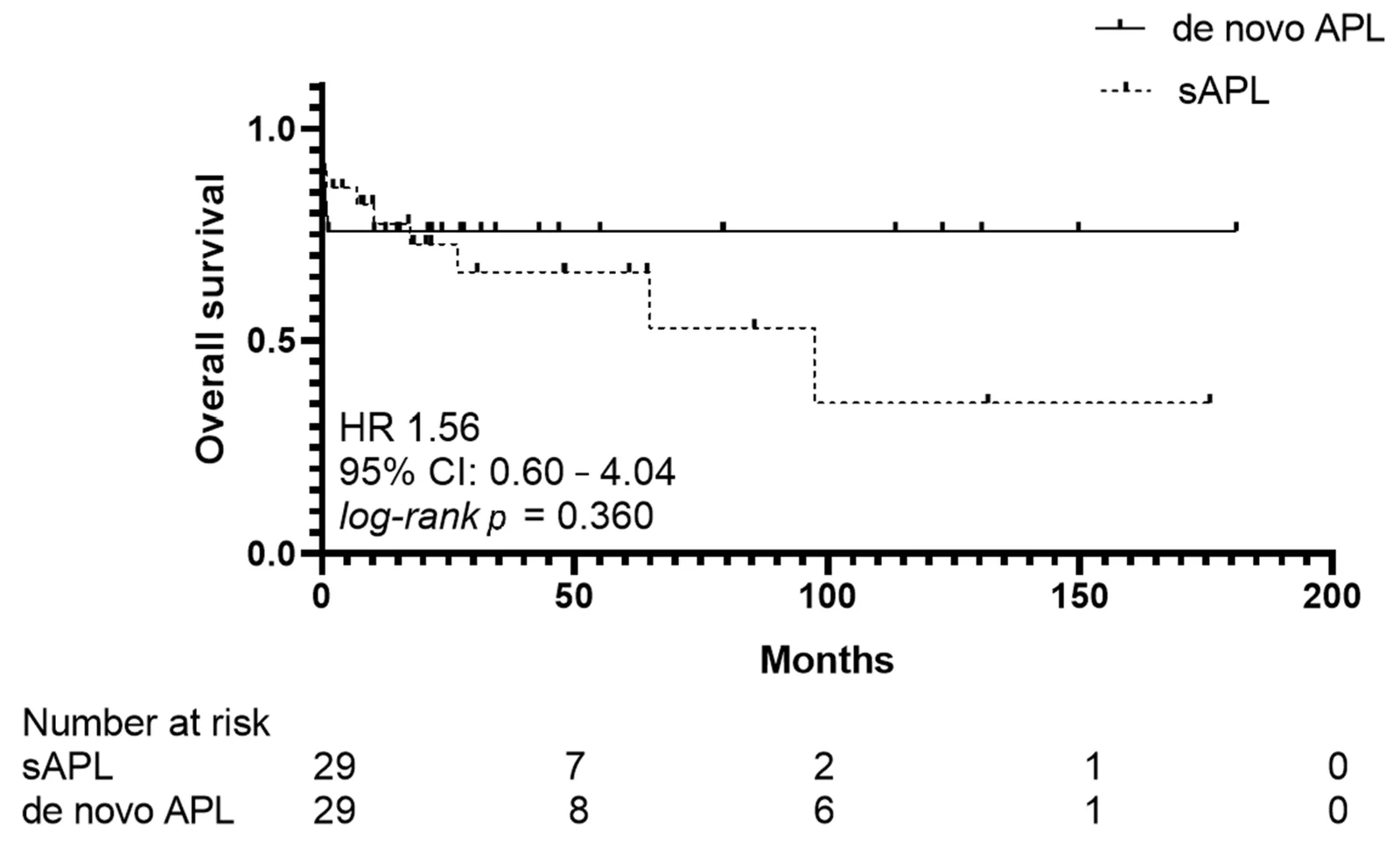
A comparison of overall survival (OS) between secondary APL and de novo APL. APL—acute promyelocytic leukemia; sAPL—secondary APL.

The median OS was 97.4 months in the sAPL group (95% CI: 26.9-NR), while median OS was not reached in the de novo APL group (*p* = 0.360; Figure 2) nor in the group as a whole.

**Figure 3:**
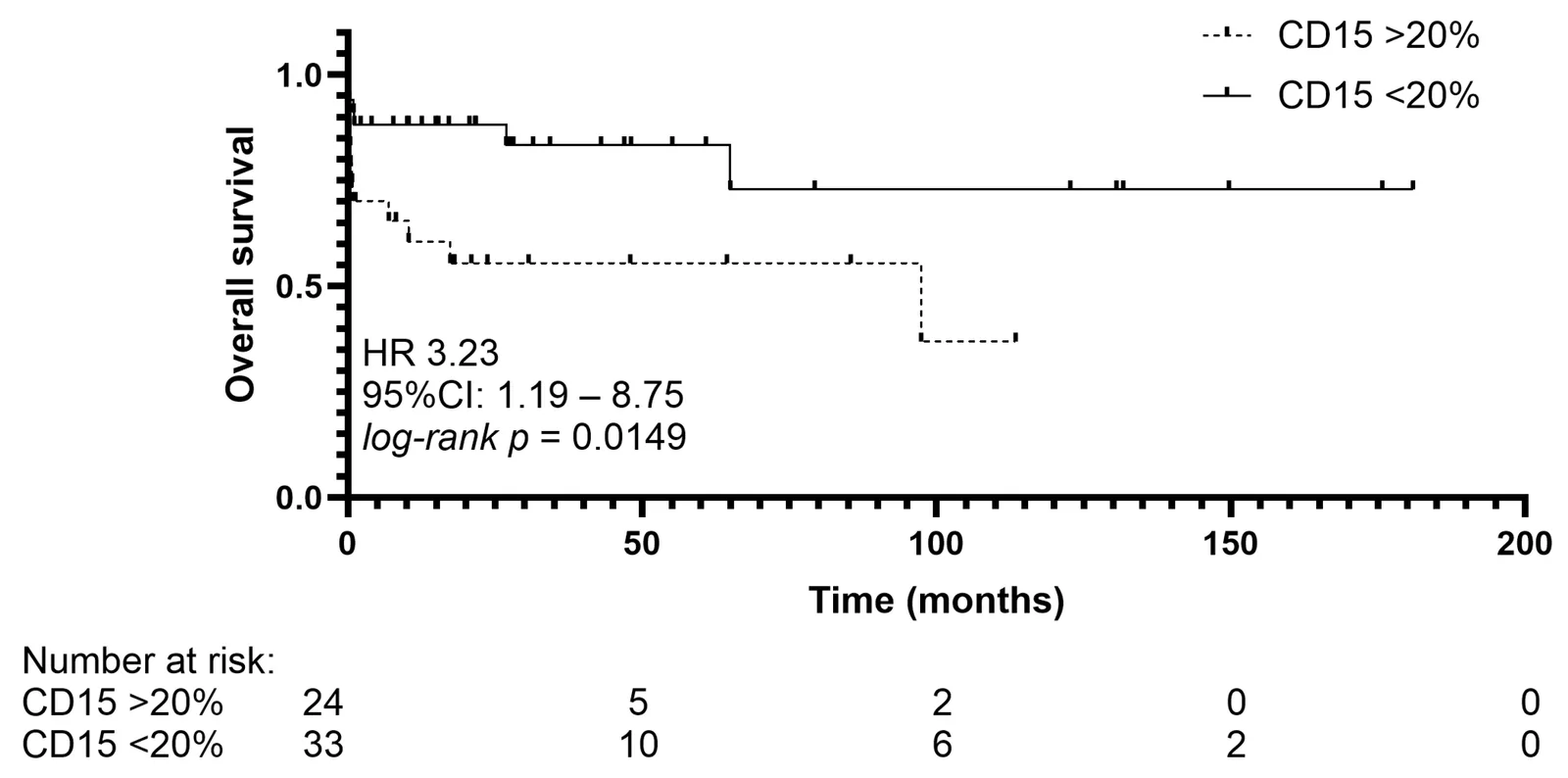
A comparison of overall survival (OS) between APL patients with and without >20% CD15 expression.

In the univariable analysis, inferior OS was associated with high-risk APL according to the Sanz score (HR 3.76, 95% CI 1.44–9.81; *p* = 0.0067), ECOG performance status ≥ 3 (HR 8.06, 95% CI 3.01–21.61; *p* < 0.001), grade 4 thrombocytopenia (HR 3.08, 95% CI 1.14–8.30; *p* = 0.0264), and CD15 > 20% (HR 3.23, 95% CI: 1.19–8.75; *p* = 0.02) (Table 2). In the corresponding Kaplan–Meier analysis, patients with CD15 expression > 20% had significantly shorter OS (*p* = 0.015; Figure 3). In the multivariable analysis, which included the variables significant in the univariable analysis together with sAPL status, ECOG performance status ≥ 3 (adjusted HR 7.48, 95% CI 2.07–27.01; *p* = 0.0021) and CD15 expression > 20% (adjusted HR 3.53, 95% CI 1.03–12.05; *p* = 0.0443) remained independently associated with inferior OS.

## 4. Discussion

This study is one of the most extensive case-matched analyses to date comparing secondary APL with de novo APL; furthermore, an important point is that one-fifth of the included patients were treated with the ATRA-ATO regimen. Our findings offer valuable insights into the clinical and biological characteristics of sAPL, and highlight important considerations for its management and prognosis.

The source of the analysis was based on a database of 437 cases registered between 2006 and 2024. Of these, the sAPL population comprised 6.70; the group was characterized by a median age of 57 years and a female prevalence.

The two groups demonstrated comparable complete remission rates after induction, with sAPL at 82.8% and de novo APL at 75.9% (*p* = 0.75). However, the sAPL group was characterized by shorter relapse-free survival and median OS (94.7 months and 97.4 months, respectively) than the de novo APL group: relapse not reached (*p* = 0.030) and median OS not reached (*p* = 0.360). In the univariate analysis, high-risk APL according to the Sanz score, ECOG ≥ 3, grade 4 thrombocytopenia, and CD15 > 20% were associated with inferior OS. In the multivariate analysis, adjusted for sAPL/de novo APL diagnosis, only ECOG ≥ 3 and CD15 > 20% were independent predictors of shorter OS.

The analyses of cases registered in the US SEER database between 2000 and 2014 identified 3877 patients with APL with a median age of 68 years; this group included 465 patients (11.0%) with sAPL [18]. The incidence of both de novo and sAPL is determined by population type, with individuals of Spanish ancestry being more prone to APL than other populations [3,4,18,19], and the Polish population, which is relatively homogeneous, having a lower APL rate than the European average (0.023 vs. 0.112 per 100,000 population per year) [3]. The profile of previous cancers noted in the studied population is comparable to that noted in previous studies, with a frequency similar to cancers found in the general population [18,19].

Our results are in line with previously published data indicating that sAPL presents similar biological characteristics, hematological findings, and cytogenetic abnormalities, to de novo APL [6,9,18,19,20]. This contrasts with sAML, which demonstrates different karyotypic aberrations to de novo AML [1,2].

Our analysis found sAPL and de novo APL to have broadly similar baseline clinical and laboratory characteristics, suggesting that in both cases, a pivotal role in the underlying leukemogenic mechanism may be played by the *PML-RARα* fusion gene. However, certain trends may warrant further investigation. Notably, sAPL patients tended to have more severe thrombocytopenia and poorer performance status (ECOG ≥ 3), which, although not statistically significant, may reflect more advanced disease biology or the cumulative effects of prior therapies. Unfortunately, it was not possible to compare our findings with other published data, as those studies include only epidemiological features. However, the poorer performance status and the fact that the disease was more advanced at diagnosis may be associated with exhaustion resulting from the previous neoplasm and its treatment.

CD15 is a differentiation-linked antigen, also known as Lewis X antigen, and is typically expressed on neutrophils and other mature myeloid cells. It plays a role in adhesion and phagocytosis [21]. During normal myelopoiesis, CD15 expression rises as progenitors mature toward neutrophils and monocytes, and its appearance is actively regulated by sialidase-mediated desialylation of precursor glycans on the cell surface [22,23]. Moreover, CD15 functions as a ligand for E-, P-, and L-selectin on vascular endothelium. This adhesion function enables leukocyte rolling and transendothelial migration in normal immunity [24]. CD15 expression supports egress from and re-entry into the bone marrow niche and may protect leukemic cells within stromal niches that are relatively shielded from chemotherapy [24]. In AML, high CD15 expression correlates with increased extramedullary infiltration, higher relapse rates, and poor survival, and is considered a marker of minimal residual disease [24]. In APL, however, unlike other AML subtypes, CD15 expression is usually absent or only weakly expressed, in contrast to other AML subtypes [24]. It is, therefore, interesting that CD15 expression was found to be significantly higher in the sAPL patients, and that it was identified as an independent predictor of inferior overall survival in our multivariate analysis. This suggests that CD15 may serve as a potential prognostic biomarker in sAPL, although this warrants further validation in larger cohorts. Previous analyses have shown CD15 expression to have potential prognostic implications; Breccia et al. [25] report high CD15 expression to be linked to an increased risk of relapse and shorter survival in 114 patients with APL. Moreover, Kutyna M. et al. [26] showed that prior cytotoxic treatment may also modify the bone-marrow microenvironment. Stromal cells from patients with therapy-related myeloid neoplasms have been observed to exhibit cellular senescence, impaired DNA-damage repair, reduced hematopoietic-supporting capacity, and a pro-inflammatory secretory phenotype following cytotoxic exposure [26]. Increased CD15 expression in sAPL may therefore represent a surrogate of leukemia-intrinsic differentiation changes, adaptation to a therapy-altered bone-marrow niche, or both. However, these interpretations remain hypothesis-generating because functional, molecular, and microenvironmental analyses were not available in the present study.

It is important to note that, despite similar rates of remission and early deaths, our sAPL patients exhibited a significantly shorter relapse-free survival (RFS), with a hazard ratio of 7.23, compared to those with de novo APL. This finding underscores the possibility that while initially responsive to initial treatment, sAPL may harbor intrinsic or acquired resistance mechanisms that increase the risk of earlier relapse.

Interestingly, overall survival (OS) did not significantly differ between groups, although the median OS was numerically shorter in the sAPL cohort. It should, however, be noted that the study included only a relatively small number of patients, and hence the analysis may have limited power to detect statistically significant differences. Nevertheless, these results suggest that while initial remission is achievable in sAPL, long-term disease control remains more challenging.

This study is not without limitations. Firstly, its retrospective design, small number of sAPL cases, and differences between treatment eras restrict the generalizability of our findings. Additionally, some data on treatment variables, maintenance completion, dose intensity, treatment interruptions, reasons for premature discontinuation, and long-term follow-up were not available consistently across participating centers and may affect the robustness of survival estimates. Nevertheless, the matched design and real-world data included in the study allow a meaningful comparison to be made of the groups and generate important hypotheses for future investigation. Further research is needed to elucidate the molecular underpinnings of sAPL, determine whether it constitutes a distinct biological subtype, and identify therapeutic strategies aimed at reducing the risk of relapse.

## 5. Conclusions

sAPL shares many clinical features with de novo APL and responds similarly to modern frontline therapies, including AIDA and ATRA-ATO; however, the condition is associated with a significantly shorter relapse-free survival, indicating a potentially more aggressive disease course. CD15 expression and poor performance status are key prognostic factors that may guide future risk-adapted treatment strategies.

## Figures and Tables

**Table 1:** Comparison of basic clinical and laboratory characteristics between sAPL and de novo APL cohorts. Data are presented as median (interquartile range, IQR) for continuous variables and as frequencies (%) for categorical variables.

Variable	sAPL	De Novo APL	*p*-Value
Age years	57 (44–60)	54 (47–63)	0.97
Sex *n* (%)	F: 18 (62%) M: 11 (38%)	F: 18 (62%) M: 11 (38%)	1.00
Sanz risk group *n* (%) Low Intermediate High Missing data	11 (38%) 10 (35%) 7 (24%) 1 (3%)	9 (31%) 13 (45%) 7 (24%) 0 (0%)	0.75
ECOG ≥ 3 *n* (%)	6 (22%)	2 (7%)	0.14
WBC (G/L)	2.5 (1.03–20.8)	1.8 (0.9–10.1)	0.66
HGB (g/dL)	9.6 (8.15–10.8)	10.1 (8.5–10.6)	0.34
PLT (G/L)	26.5 (16–54)	37 (25–57)	0.19
Fibrinogen (g/L)	1.4 (0.42–2.6)	1.41 (0.91–2.8)	0.72
D-dimer (ug/mL)	35.2 (15.3–49.5)	19.8 (3.51–56)	0.43
LDH (IU/L)	327 (179–387)	422 (173–759)	0.80
PT (s)	12.9 (10.2–20.6)	14.6 (12.2–16)	1.00
APTT (s)	26.8 (18.3–31.7)	27.7 (26.8–32)	0.32
BM blasts (%)	75.5 (50–82)	78.5 (56- 89)	0.54

WBC—White blood cell count, HGB—hemoglobin, PLT—platelets, LDH—lactate dehydrogenase, PT—prothrombin time, APTT—Activated Partial Thromboplastin Time, BM—bone marrow.

**Table 2:** Univariable and multivariable Cox proportional hazards regression analyses of overall survival in the entire APL cohort. Variables significant in the univariable analyses were included in the multivariable model together with sAPL status.

Variable	*p* Value	HR	95% CI		*p* Value	HR	95% CI	
			Lower	Upper			Lower	Upper
sAPL	0.36	1.57	0.59	4.13	0.19	0.40	0.11	1.55
Age at APL diagnosis *	0.34	1.02	0.98	1.06				
High-risk APL	0.006	3.76	1.44	9.81	0.19	2.22	0.67	7.31
ECOG 3/4	<0.01	8.06	3.01	21.61	<0.01	7.48	2.07	27.01
Percentage blasts in BM *	0.25	0.99	0.96	1.01				
Hemoglobin * (g/dL)	0.57	1.00	1.00	1.00				
WBC * (G/L)	0.54	1.00	1.00	1.00				
Thrombocytopenia grade 4	0.03	3.08	1.14	8.30	0.19	2.37	0.66	8.53
Fibrinogen * (g/L)	0.32	0.62	0.24	1.60				
CD15 > 20%	0.02	3.23	1.19	8.75	0.04	3.53	1.03	12.05
Additional cytogenetic aberrations	0.92	0.95	0.33	2.74				
FLT3-ITD	0.64	0.91	0.18	4.68				

* HR, hazard ratio; CI, confidence interval, sAPL, secondary acute promyelocytic leukemia, BM, bone marrow, WBC, white blood cell. The reference categories were de novo APL, low/intermediate-risk APL, ECOG performance status 0–2, absence of grade 4 thrombocytopenia, and CD15 expression ≤ 20%, as applicable.

## Data Availability

The original contributions presented in this study are included in the article/Supplementary Materials. Further inquiries can be directed to the corresponding author.

## Supplementary Materials

The following supporting information can be downloaded at: https://www.mdpi.com/article/10.3390/cancers18182899/s1, Figure S1: Comparison of CD15 expression between blasts. In sAPL, significantly higher CD15 expression was found (median 27.56%, IQR: 2.43–51.50 vs. 7.00%, IQR: 0.62–26.00, *p* = 0.04). APL—acute promyelocytic leukemia; sAPL—secondary APL.
