# Supplementary material for: A Comparison of Outcomes in Patients with Secondary Acute Promyelocytic Leukemia (APL) and De Novo APL—A Case-Matched Retrospective Analysis of the Polish Adult Leukemia Group (PALG)
Source: Cancers (Basel). 2026 Sep 8;18(18):2899. doi: 10.3390/cancers18182899 (PMC13604275; doi:10.3390/cancers18182899)
Supplement: Supplementary file 1 [file cancers-18-02899-s001.zip › cancers-4513163-supplementary.pdf]

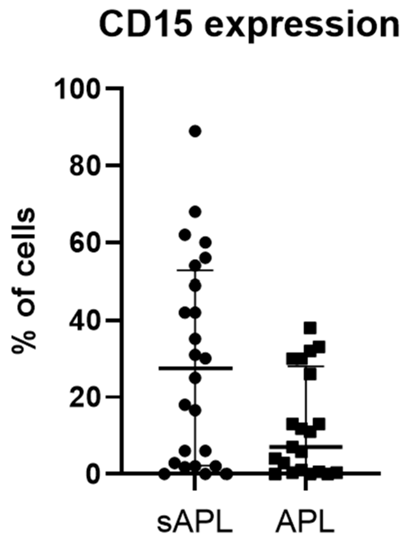


**Supplementary Figure S1**. Comparison of CD15 expression between blasts. In sAPL, significantly higher CD15 expression was found (median 27.56%, IQR: 2.43–51.50 vs. 7.00%, IQR: 0.62–26.00, $p$ = 0.04). APL—acute promyelocytic leukemia; sAPL—secondary APL.
